# Preparation of Microemulsion from an Alkyl Polyglycoside Surfactant and Tea Tree Oil

**DOI:** 10.3390/molecules26071971

**Published:** 2021-03-31

**Authors:** Thuy-Vi Vo, Ya-Yen Chou, Bing-Hung Chen

**Affiliations:** 1 Department of Chemical Engineering, National Cheng Kung University, Tainan 70101, Taiwan; vivt@hufi.edu.vn; 2 Department of Leisure and Sports Management, Far East University, Tainan 74448, Taiwan; f44@ms21.hinet.net

**Keywords:** microemulsion, tea tree oil, alkyl polyglycoside, hydrodistillation, humectant

## Abstract

Preparation and characterization of microemulsions consisting of a plant-derived alkyl polyglycoside (APG) surfactant and the essential oil of *Melaleuca alternifolia* (tea tree) was studied. This nonionic APG surfactant used was Triton CG-110 with a CMC at 1748 ppm at 25 °C. Tea tree oil (TTO) was extracted from tea tree leaves by Triton CG-110-assisted hydrodistillation method. The preparation of the microemulsion was aided by the construction of pseudo-ternary phase diagrams, which were investigated at the different weight ratios of surfactant mixtures (S_mix_ = Triton CG-110/PPG) as 0.6:1, 1.8:1, 1:0 with hydrodistilled and commercial TTO by water titration method at room temperature. Particularly, structure of microemulsion was identified by electrical conductivity and viscosity. Moreover, shelf stability of some microemulsion made of 1% TTO with various concentration of Triton CG-110/PPG (1.8:1 *w*/*w*) were monitored for over a two-month period with dynamic light scattering. These results showed that microemulsion made of 1% TTO, 9% Triton CG-110/PPG (1.8:1 *w*/*w*) was insensitive with time and temperature of storage.

## 1. Introduction

Alkyl polyglycosides (APGs) are an emerging class of nonionic surfactants derived from renewable plant-based materials [1,2,3,4]. In general, the starting raw materials of APGs are typically glucose, a fraction of degraded starch, and fatty alcohols [1,2,3,4]. APGs are commonly synthesized either by acetalization of glucose with fatty alcohols or by transacetalization of short-chain alkyl glucosides with long-chain alcohols in presence of acid catalysts is used in both processes [1,2,3,4]. Though the first APG was synthesized by Emil Fischer more than a century ago, the industrial production of APGs as surfactants was commenced not until 30 years ago, namely since early 1990’s [1,2,3,4]. Notably, APGs are made by combining vegetable oils and sugar as raw materials and, hence, regarded as the first kind of surfactants commercially available that are completely based on renewable sources [1,2,3,4]. In contrast to conventional nonionic surfactants which are mostly given with hydrophilicity from polyoxyethylene units on surfactant molecules, the hydrophilic nature of APGs is resulted from their glucoside moieties. 

Even though the hydrophilic moieties of APG molecules are mainly oligosaccharides or oligoglycosides, APGs are often classified according to the alkyl chain length of their hydrophobic groups. For example, coco glucoside and lauryl glucoside, resulted from the condensation of plant-derived glucose with coconut alcohol and lauryl alcohol, contain similar length of alkyl chains but differ significantly in physicochemical properties [5]. Besides, stereoisomer with α and β-epimers and ring isomers in pyranoside and furanoside forms are commonly adopted to identify APGs. In principle, both the chain length of the hydrophobic alkyl groups and the degree of glucosidation are considered as key parameters to determine the amphiphilicity of APGs and, therefore, the phase and aggregation behaviors of APGs [2,3,4].

APGs are known to possess low toxicity and are readily biodegradable. With the favorable and excellent ecotoxicological profiles, they have been regarded as biodegradable greener surfactants [1,2,3,4]. APGs with long alkyl chains, e.g., C_8_/_10_- and C_12_/_14_-alkyl polyglycosides, have recently been adopted in the formulation for cosmetics/personal-care products, household and industrial and institution (I&I) detergents, highly alkaline detergents, glass cleaners, metal cleaners agrochemicals, pharmaceuticals, etc. [1,2] Applications of APGs in personal care products are mainly based on their superior foaming and dermatological compatibility, as well as lower skin irritation ability [2,3,4,6]. The alkyl polyglucoside surfactant with the trademark as Triton CG-110 used in this work was fabricated by etherification of polyglucose containing from 2 to 6 glucose units with a mixture 50/50 of octanol and decanol [7]. However, the study on the applications of Triton CG-110 as microemulsion carriers for essential oils for personal care applications is rarely found in the open literature [6]. Hence, it is our attempt to explore the feasibility of using such a plant-derived APG to form microemulsions with a model essential oil, in which Australian tea tree oil was chosen as the model oil.

Tea tree oil (TTO), the volatile essential oil extracted from the *Melaleuca alternifolia*, has been popularly marketed from Australia all over the world since 1920s [8,9]. It is abundantly supplied over the counters in Australia, Europe, and North America. Moreover, TTO is generally accepted and used as a traditional herbal medicine, especially for antibacterial, antifungal, antiviral and anti-inflammatory actions in cutaneous applications [6,8,9,10,11]. The vital usages are also found in treatment of acne, skin related disease, oral candidiasis, and even prevention from mosquito-borne diseases [12,13,14,15,16]. Notably, surfactant aggregates, especially microemulsions, were adopted as administration vehicles for the hydrophobic TTOs to serve the aforementioned applications. Moreover, with the fact on the widespread availability of TTO and a concern in adverse impacts on health owing to the excess amounts in some ingredients of TTO, the International Organization for Standardization develops and publishes the ISO 4730 standard to specify TTO and to facilitate assessment of TTO’s quality [17]. In this study, TTO was extracted from fresh tea tree leafs by the Triton CG-110 surfactant-assisted hydrodistillation method. The in-house extracted TTO was in good compliance with ISO 4730.

IUPAC defines microemulsion as isotropic, thermodynamically stable colloidal systems made of water, oil and surfactant which spontaneously formed with aggregate size found in the range of 1–100 nm, usually from 10 to 50 nm [18,19,20,21]. In brief, microemulsions are thermodynamically stable dispersions of oil and water made with surfactant molecules, frequently in combination with co-surfactants [19,22,23,24]. Meanwhile, micelles could be swollen by oils, in resemblance with microemulsions, and often referred as “swollen micelles” [22]. In general, microemulsions are regarded as aggregates larger than micelles. Typically, the o/w microemulsions possess a higher encapsulation efficiency of oil molecules than normal micelles [6,11,14,25,26]. For example, microemulsions prepared from TTO with sucrose laurate and propylene glycol exhibited excellent shelf stability and retained superior biological activity and antiradical activity [25]. Furthermore, the microemulsions made of up to 0.5 wt% tea tree oil with 2 wt% Polysorbate 80, aka Tween 80 remained stable more than one month [26].

In microemulsions, polar compounds of intermediate chain length as cosurfactants are often added in the colloidal dispersion to provide the proper balance between hydrophilic and lipophilic properties for the required oil and water phases [19,22,23,24]. Generally, co-surfactants are short-chained alcohols and amines. Importantly, the chain length of cosurfactant molecules is significantly different from that of surfactant molecules. Under circumstances with balanced hydrophilicity and lipophilicity, interpolation of cosurfactant molecules into the interfacial films of oil-surfactant or water-surfactant would result in random misalignment of palisade layers of surfactant aggregates and, consequently, lower the bending energy of surfactant films or increase the film elasticity [22,23]. Hence, an ultra-low interfacial tension could be attained with addition of proper co-surfactants [22,23].

In personal care products, humectants are hydroscopic organic compounds and, generally, formulated in microemulsions especially for topical applications. Common humectants include polymeric polyols, e.g., polyethylene glycol (PEG), short-chain aliphatic polyols (e.g., propylene glycol, glycerol and butylene glycol), urea, lactic acid, sodium pyrrolidone carboxylate (Na-PCA), etc. In this work, propylene glycol (PPG) and glycerol, adopted as both humectants and cosurfactants, were studied for their influence in the TTO-APG-based microemulsions. To provide a comparison with the effects of PPG and glycerol on microemulsions, PEG 400, a common humectant but never a cosurfactant, was adopted for the study on its role in microemulsions as well. Notably, because of its viscous characteristics, PEG 400 was often utilized as a viscosity enhancer in cosmetics, in addition to the use as a humectant.

In brief, this study focused on the applications of Triton CG-110 to form microemulsions with tea tree oil with an aim to improve the apparent solubility of tea tree oil in aqueous media. Furthermore, propylene glycol, glycerol and PEG 400 were chosen for the study on the effect as humectants and co-surfactants in the resultant TTO-based microemulsions. In practical approaches, pseudo-ternary phase diagrams with Triton CG-110/humectant (cosurfactant) mixtures and TTO in water were constructed. Subsequently, the compositions of microemulsion phase could be located on these ternary phase diagrams. The resultant microemulsions were further characterized with the measurements of electrical conductivity, viscosity, and particle size by dynamic light scattering technique, as well as with the morphology observed on the transmission electron microscope.

## 2. Results

### 2.1. The Compositions of the Tea Tree Oil

GC-FID analyses and GC-MS analyses were both applied to identify nine major compounds in tea tree oil (Table 1). An internal standard, *n*-decane, was used to quantify these individual components in the hydrodistilled tea tree oil. Terpinen-4-ol was found with the most ample ingredient, also specified as the primary TTO component in the ISO 4730:2017 Essential oil of *Melaleuca*, terpinen-4-ol type (Tea Tree oil) [17]. Other components could be assigned to *α*-pinene, sabinene, β-pinene, *α*-terpinene, *p*-cymene, limonene, 1,8-cineole, *γ*-terpinene, terpinolene, *α*-terpineol, cadinene, globulol, and cubenol, according to the GC-MS analyses with a reference to those listed in the NIST library. In this work, Triton CG-110 with a concentration as 650 ppm was used to assist the extraction of TTO by hydrodistillation. Interestingly, the extraction efficiency of tea tree oil by hydrodistillation was improved by 10% in presence of 650 ppm Triton CG-110 in extraction media. However, no trace of Triton CG-110 was found in the as-extracted TTO. This could be attributable to the high boiling point of Triton CG-110, compared to those of the individual ingredients in TTO. That is, Triton CG-110 would not be conjointly hydrodistilled out to the collected TTO phase.

The compositions of the in-house hydrodistilled TTO and the commercial TTO were all tabulated Table 1. Notably, those nine main constituents made up to 89.8% of commercial TTO sample tested and 89.1% in the in-house hydrodistilled oil. Especially, terpinen-4-ol was measured as 43.0% in commercial oil and 47.4% in our hydrodistilled oil, which were both in good compliance with the ISO 4730:2017. Meanwhile, the content of the irritating eucalyptol, i.e., 1,8-cineol of the in-house hydrodistilled TTO was 3.6%, lower than 10% which was cautiously emphasized by ISO 4730:2017 as the maximum allowable in TTO.

### 2.2. Phase Behavior of Tea Tree Oil (TTO)—Mixed Surfactants—Water

**Figure 1:**
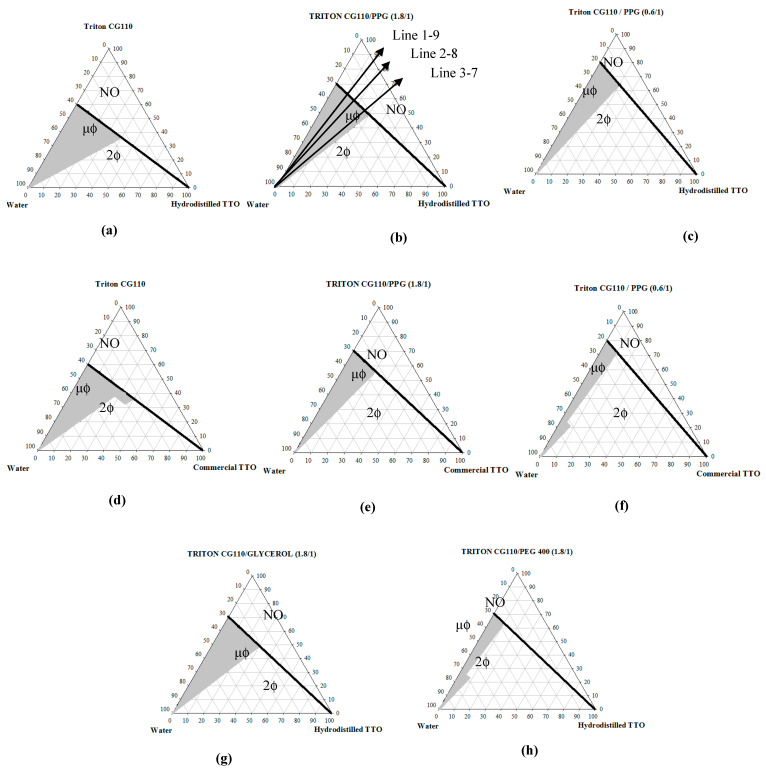
Pseudoternary phase diagrams of mixed surfactant—tea tree oil (TTO)—water systems. The mixed surfactants were prepared from Triton CG-110 and a co-surfactant at different mass ratios (either 0.6:1 or 1.8:1). The co-surfactants include glycerol, propylene glycol (PPG) and polyethylene glycol 400 (PEG 400), while either in-house hydrodistilled TTO or commercial TTO was used. (**a**,**d**) Triton CG-110 only, (**b**,**e**) Triton CG-110/ PPG (1.8/1), (**c**,**f**) Triton CG-110/PPG (0.6/1), (**g**) Triton CG-110/glycerol (1.8/1), (**h**) Triton CG-110/PEG 400 (1.8/1).

The pseudo-ternary phase diagrams of TTO-mixed surfactant-water were studied and shown in Figure 1a–g. Both TTOs, in-house hydrodistilled (Figure 1a–c,g,h) and commercially available (Figure 1d–f) were used in the phase study. Mixed surfactants were made from the mixtures of Triton CG-110 and co-surfactant/humectant in various ratios. As to be given with details in Section 4.3, Line 1–9, Line 2–7 and Line 3–7 represented the ratio of mixed surfactant and tea tree oil. For example, Line 1–9 was corresponding to water dilution series of the formula containing 10% oil and 90% mixed surfactant, and shown as the arrow in the Figure 1. Phase behavior was recorded based on observations of all Lines x–y. Phase diagrams shown on Figure 1 were generally divided into three regions, such as one-phase region (μϕ), two-phase region (2ϕ) and non-observable region (NO). The one-phase region, either the oil-free micellar phase or the oil-containing microemulsion phase, was painted in gray on the phase diagrams. In general, micelles and microemulsions could be formed regardless of co-surfactants. As aforementioned, the water content in the “neat” Triton CG-110 surfactant was almost 40 wt%. Thus, even samples prepared with only the commercial Triton CG-110 and oil for phase observation contained inevitably a certain amount of water. As result, the dark line separating the non-observable region (NO) and the one-phase (μϕ)/two-phase regions (2ϕ) on these phase diagrams marked the least phase compositions of samples that could be prepared with the commercial Triton CG-110 surfactant. The two-phase (2ϕ) region appeared below the black line, where the phase separation occurred owing to the limited immiscibility between TTO and mixed surfactant solution. The polarized light microscopy was employed to observe the difference between the microemulsion phase and the possibly coexisting liquid crystalline phase. Interestingly, no liquid crystalline phase was observed in all TTO microemulsions in this study. The phase diagram indicated, in general that the composition phase of microemulsion was getting wider and wider with more Triton CG-110 used in the preparation of microemulsion. Notably, no matter if with or without humectants/co-surfactants, such as propylene glycol (PPG), glycerol and polyethylene glycol 400 (PEG 400), Triton CG-110 alone could still form microemulsions with TTO in aqueous solution.

### 2.3. Microstructural Transition of Microemulsion

Among all microemulsion regimes shown on the phase diagrams of pseudoternary systems, the microemulsion made from mixed surfactant (Triton CG-110/PPG = 1.8/1 by mass) and tea tree oil possessed relatively wider ranges on these phase diagrams. Consequently, microemulsion prepared from this system was further characterized with various instruments for their physical properties, including electrical conductivity, viscosity and hydrodynamic size.

**Figure 2:**
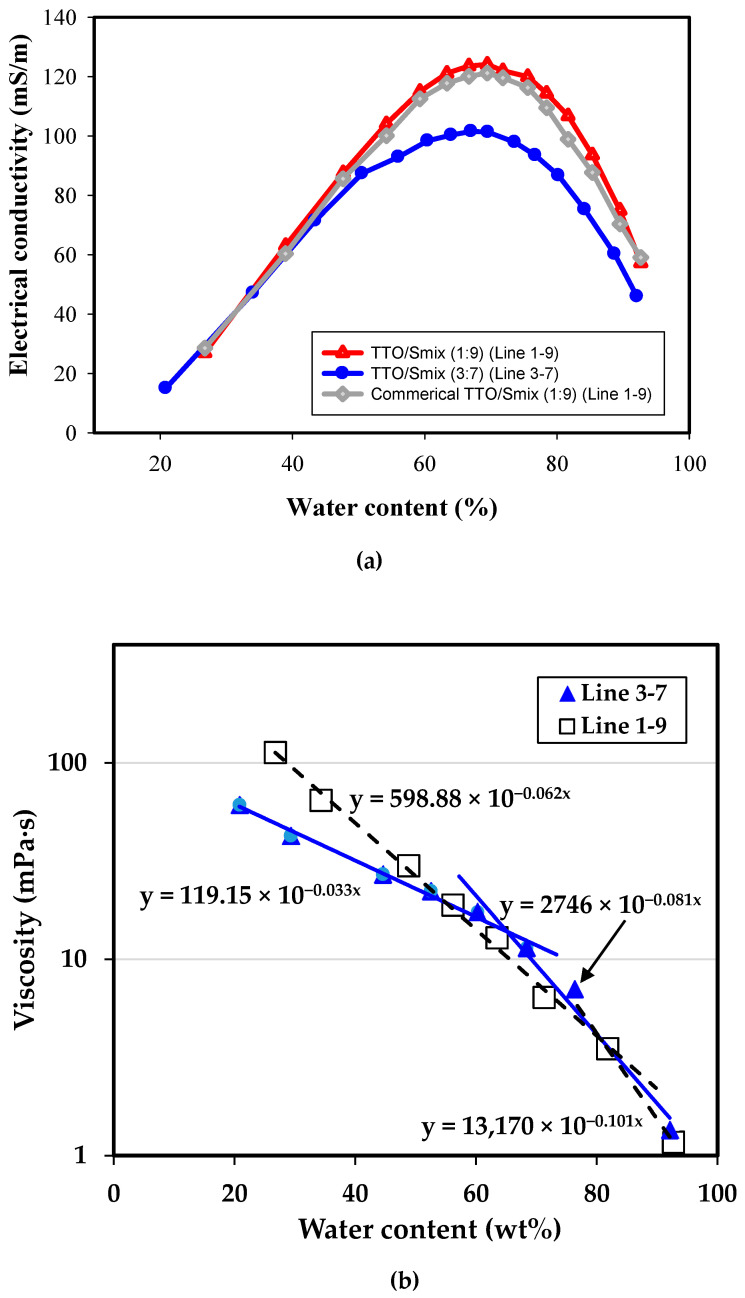
Properties of microemulsions prepared from dilution along Line 1–9 and Line 3–7: (**a**) Electrical conductivity; (**b**) Viscosity.

Conductivimetry is a common tool to examine the phase structures of ionic surfactant aggregates, e.g., the critical micelle concentration and the phase transition of microemulsion [25,26,27]. Therefore, electrical conductivity (κ) of microemulsion made with Triton CG-110/PPG in a ratio 1.8/1 was measured along the water dilution lines, Line 3–7 and Line 1–9 (Figure 2a). The electrical conductivity was plotted against the actual water content (Φ_W_) of microemulsion, which was the total amount of water including that intrinsically found in neat Triton CG-110 surfactant (i.e., 39.65%) and the water added to form microemulsion. That is, the minimal water contents (Φ_w_) in microemulsions of Triton CG-110/PPG (S_mix_ = 1.8) on dilution Line 3–7 and Line 1–9 were 20.8 wt% and 26.8 wt%, respectively. In general, the bell-shaped curve was observed on the electrical conductivity of microemulsion as a function of its water content.

According to the manufacturer, Triton CG-110 is nonionic. However, the pH of 5 wt% Triton CG-110 solution is 5.7, weakly acidic, instead of near 7. Its weakly acidic nature is probably attributable to the existence of ionic impurity or the carboxylic acids on surfactant molecules. Additionally, electrical conductivity could be probably resulted from the self-ionization of monoterpene alcohols and the mobility of remaining polar molecules in TTO [25]. Therefore, prior to water titration, the TTO/Smix mixtures along Line 3–7 and Line 1–9 presented the *κ* values of 15.1 mS/m and 27.0 mS/m, respectively. The *κ* value of the resultant microemulsion increased linearly with an increasing amount of water till Φ_w_~55% (Figure 2a). Meanwhile, the color of microemulsion along Line 3–7 turned translucent, while that of microemulsion was still yellowish. The slope of electric conductivity as a function of water content varies with different surfactant aggregates [25,26,27]. Hence, the fact of the uniform slope of *κ* values vs. water contents shown in Figure 2a implied no discernible sign in phase transition from W/O microemulsion to bicontinuous (cubic) phase and from bicontinuous phase to O/W microemulsions was found in this work, in contrast to those reported in the literature [25,26,27]. W/O microemulsion would be expected to form only in low-water region. However, the minimal water contents in microemulsions prepared in this work was greater than 20 wt%, which could be too large to observe the formation of W/O microemulsion. Alternatively, the increment in electric conductivity could be resulted from more dissociation of carboxylic acids from surfactants.

Upon further dilution with water, the *κ* values increased gradually with the corrected water contents from 55 wt% to 70 wt%, and reached the maximum at 69.5 wt%. The *κ* value of the microemulsion decreased again sharply and linearly with the further increasing water content. The decrease in conductivity arose merely from the dilution of surfactant in microemulsions, instead of transition from bicontinuous phase to O/W microemulsion [25]. The dominating structure before the maximum conductivity could be the O/W microemulsion [28,29]. Similarly, in this decreasing conductivity region, the surfactant molecules became saturated with water molecules and the oil-swollen micellar phase was developed [22]. In general, microemulsion along Line 3–7 has less surfactant in formulation and, thus, its conductivity is lower than that of microemulsion along Line 1–9. Interestingly, though tea tree oil is a mixture of nonionic species, the conductivity of microemulsion along Line 1–9 made with commercially acquired TTO is mostly less than that made with in-house hydrodistilled TTO and the same amount of water, as shown in Figure 2a.

Rheological measurements on selected microemulsion samples made along Line 1–9 and Line 3–7 were carried out with a flow sweep from 300 s^−1^ to 1 s^−1^. The lowest shear rate for samples to yield reliable measurements was 1 s^−1^. In general, Newtonian behaviors were observed on these microemulsion samples. The dependence of the dynamic viscosity (η) of microemulsions on their corrected water contents along both dilution lines were measured at a shear rate of 10 s^−1^. The apparent viscosity of microemulsion on Line 3–7 without addition of external water was 61 mPa·s. Notably, the actual water content in this sample was 20.8 wt%. Similarly, the microemulsion sample made from Line 1–9 with addition of water, i.e., the true water content equal to 26.8 wt%, had an apparent viscosity 112 mPa·s. With a further dilution of microemulsion, the viscosity of the microemulsion was decreased obviously. An empirical power-law could be derived to describe the viscosity of the microemulsion as a function of its water content, shown as follows:

(1) η=A·exp(B·ω)

where *η* is the viscosity (mPa·s), *ω* the water content in wt%, and *A* as well as *B* the constants. Interestingly, the viscosity of the microemulsion obtained from Line 3–7 required two sets of parameter A and power index B to yield a good fit with R_2_ > 0.97. The power index changed significantly from B = −0.033 in lower-water region to B = −0.081 for diluted region. The turning transition on the trend of concentration dependent viscosity occurred at a water content near 65 wt%, coincidently with the water content where the maximum conductivity of microemulsion was found on Figure 2a. Similarly, the viscosity of the microemulsion made from Line 1–9 could be fit well with two set of the coefficient A and the power index B (Figure 2a). Again, the transition in viscosity behavior of the microemulsion took place at the water content at 79.2 wt%, in the proximity of the maximum conductivity.

### 2.4. Shelf Stability of Microemulsion

**Figure 3:**
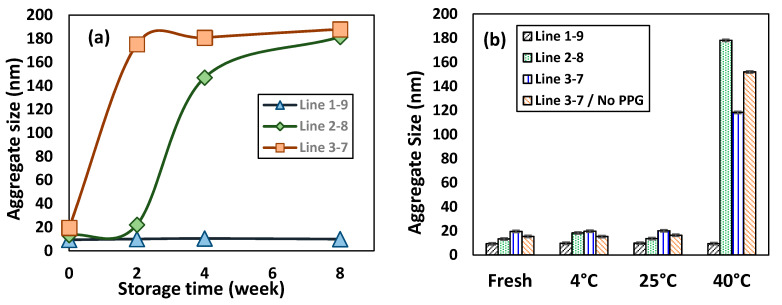
Effect of storage temperature and time on the aggregate size of microemulsion prepare from mixed surfactants of Triton CG-110/PPG (1.8:1 *w*/*w*) and 1 wt% *in-house* hydrodistilled TTO: (**a**) Stored at 25 °C, (**b**) Measured after one-week storage.

Shelf stability of microemulsions was monitored mainly by the size of the microemulsion droplets measured with dynamic light scattering (DLS) technique, as shown in Figure 3. These microemulsion samples were prepared along dilution Line 1–9, Line 2–8 and Line 3–7 using the mixed surfactant of Triton CG-110/PPG in a mass ratio 1.8/1 (S_mix_ = 1.8), while keeping TTO concentrations constant at 1 wt% in these samples. That is, the actual concentrations of Triton CG-110 in these microemulsion samples were 4.07 wt%, 1.81 wt% and 1.06 wt%, respectively, in those made along Line 1–9, Line 2–8 and Line 3–7. Figure 3a showed the change on the average sizes of microemulsions over a storage period of two months. These samples were stored in an air-conditioned room at 25 °C. The size of fresh microemulsions was found from 9.3 nm for that made with Line 1–9 to 19.5 nm for that from Line 3–7, comparable to those reported from previous studies [25,26]. Alternatively, the aggegate size increased from 9.3 nm to 19.5 nm as surfactant concentration decreased from 5.79 wt% to 1.50 wt%. More Triton CG-110 molecules would facilitate solubilization of TTO into water to form more compact aggregates. The droplet sizes of these three micromulsions dis not change obviously over a week stored at 25 °C. However, with an increasing storage time further to two weeks, the aggegate size of microemulsion made from Line 3–7 increased significantly to ca. 175 nm, while the other two remained at about 22 nm and 10 nm. It grew further to 188 nm after 8 weeks in storage. Interestingly, the microemulsion droplets made from Line 2–8 increased to 146 nm after 4 weeks in storage and, eventually, to 181 nm after 8 weeks. In contrast, the aggregate size of microemulsion made from Line 1–9 remained almost constant near 10 nm over 8 weeks in storage at 25 °C, similar to TTO microemulsions with sucrose laurate/PPG mixed surfactant [25]. In contrast, microemulsions containing 0.25 wt% to 0.5 wt% TTO with 2 wt% Tween 80 (polysorbate 80) showed dramatical reduction in aggregate size from 93.6 nm–195 nm measured right after preparation to ca. 11 nm after a storage period of 30 days [26].

Furthermore, effect of storage temperature to aggregate size of microemulsion consisting of 1% in-house hydrodistilled TTO with various concentration of Triton CG-110/PPG (1.8:1 *w*/*w*) were examined after one-week storage at 4 °C, 25 °C and 40 °C (Figure 3b). At 4 °C or 25 °C, all microemulsion samples possess the aggregate sizes comparable to those of freshly prepared microemulsions (Figure 3b). As expected, the droplet sizes of nonionic microemulsions will increase with a higher temperature, as nonionic surfactants will lose their hydrophilicity and result in the dehydration of aggregates. Upon reaching the phase-inversion temperature, the O/W microemulsions will be transformed preferably into W/O microemulsions. At 40 °C, except the microemulsion made from Line 1–9, the aggregate sizes of microemulsions grew significantly to ca. 120–180 nm. For comparison, the stability of microemulsion made Line 3–7 but without PPG was investigated. The particle size of microemulsion increased 7 times to ca. 150 nm at 40 °C. Hence, inclusion of PPG has little effect on the aggregate size of microemulsion stored for a week at 40 °C.

Tea tree oil is a mixture of various phytochemicals, among which the terpinen-4-ol is the major component. Over the long period of storage, the relatively hydrophilic ingredients of tee tree oils could preferably solubilized into smaller micelles. Therefore, the size of the microemulsion could grow significantly and the polydispersity of the size distribution could become wider. The microemulsion could be largely stabilized, if more surfactant was initially present in the microemulsions (Figure 3).

## 3. Discussion

The pseudo-phase diagrams of mixed surfactant–TTO–water systems were constructed to provide the preparation conditions for microemulsions. As shown in Figure 1, microemulsion could still be formed with addition of humectants, such as propylene glycol (PPG), glycerol and polyethylene glycol 400 (PEG 400). However, the microemulsion regions on the phase diagram, based on the same amount of Triton CG-110, seemed to shrink with addition of humectants. Among all three humectants, PEG 400 reduced the most significantly the phase space of microemulsion on the phase diagram (Figure 1h). In contrast, PPG gave relatively larger microemulsion territory (Figure 1b). In general, PPG provided with a better storage stability in the resultant microemulsions. With PPG added, the color of microemulsions along the titration lines, i.e., Line 1–9, Line 2–8 and Line 3–7, changed from yellowish, transparent, and finally to translucent, with an increasing water content.

Interestingly, formation of microemulsion was seemingly influenced by the source of the tea tree oil (Figure 1). In general, the TTO obtained by hydrodistillation in this study led to microemulsions with a wider composition range on the phase diagram (Figure 1a–c), compared to those with the commercially acquired TTO (Figure 1d–f). This could be attributable to minor differences in components in both TTOs. For example, the hydrodistilled TTO contained more terpinen-4-ol and α-terpineol than the commercial TTO, in which terpinen-4-ol and α-terpineol in TTO were reported to be able to promote the aqueous solubility of TTO [25,30,31].

Additionally, effect of humectants as co-surfactant on the formation of microemulsion was also displayed in Figure 1b,g,h. Propylene glycol, glycerol and PEG 400 were common humectants formulated in cosmetics. Moreover, these polyols have been found in vitro with the bactericidal activity against selected microorganisms as well. In general, their presence resulted in smaller composition ranges for microemulsions. That is, the inclusion of these three polyol humectants would suppress formation of microemulsions, compared to the humectant-free Triton CG-110/TTO microemulsion. In brief, the effect of humectant on the amount of TTO solubilized in microemulsions were followed by glycerol > PPG > PEG 400. However, both glycerol and PEG 400 are relatively viscous and not easily dispensed. Therefore, in order to examine the effect of humectants on the physicochemical properties of the resultant microemulsions, the fluidic PPG was chosen to formulate the microemulsions.

Although many modern methods, such as small angle X-ray scattering (SAXS), differential scanning calorimetry (DSC) and nuclear magnetic resonance (NMR), can be used in the study of microemulsion structure, electrical conductivimetry and rheology are two conventional methods that can determine microemulsion structure accurately and rapidly. In our study, the bell-shape conductivity of microemulsion and the decrease in the viscosity of microemulsion had been both observed with an increasing water content. Moreover, the transition in the trend of the viscosity of microemulsion was found to coincide with the proximity of the maximum conductivity, i.e., the water content around 79.2 wt% for Line 1–9 and about 65 wt% for Line 3–7, marking the transition possibly from a o/w microemulsion to micelles. No existence of transition from a bicontinuous cubic phase to an o/w microemulsion was noted in this system, possibly owing to the high water content initially present in the commercial Triton CG-110.

Additionally, the dynamic light scattering (DLS) technique is a useful tool in the measurement of aggregate size of microemulsion as well as in the monitoring of its shelf stability during storage. For example, microemulsion made with 1 wt% TTO and 4 wt% mixed surfactant of Triton CG-110/PPG (1.8:1 *w*/*w*) expressed the instability with an increasing aggregate size beyond 200 nm during storage. In contrast, the o/w microemulsion made with 1 wt% TTO and 9 wt% mixed surfactant of the same demonstrated a superior shelf stability over two months with an aggregate size maintaining near 10 nm.

Although TTO is known for its high antibacterial, antifungal, antiviral and anti-inflammatory activity, it could cause the irritability on the skin, as reported in the dosage with TTO more than 1%. Hence, studies on nanoparticles containing tea tree oil at diluted concentrations but retaining its superior biological properties have been continuously developed. These nanoparticles, i.e., microemulsion or nanocapsules in combination with polymers, not only help limit the evaporation rate of TTO but also reduce the formation of oxidation products. Especially, the anti-bacterial and anti-fungal efficacy on rat pneumonia were satisfactorily reported with TTO microemulsions administered at 7 mg oil/rat by inhalation therapies [32]. In our study, a novel formulation consisting of 1 wt % TTO and 9% mixed surfactant of Triton CG-110/PPG (1.8:1 *w*/*w*) in nanoscale and with superior shelf stability can be considered in the applications of cosmetics, such as acne treatment and wound dressing, or used as a medical drug in pneumonia treatment [32].

## 4. Materials and Methods

### 4.1. Materials

The leaves and twigs of the tea tree, *Melaleuca alternifolia*, were collected from a local farm in Tainan City, Taiwan. One kind of alkyl polyglycoside (APG) nonionic surfactant, Triton CG-110 (CAS number 68515-73-1), was fabricated by the Dow Chemical Company (Midland, MI, USA) and supplied from Sigma-Aldrich (St. Louis, MO, USA). According to the manufacturer, the critical micelle concentration (CMC) of Triton CG-110 at 25 °C is 1748 ppm, while its cloud point in 1 wt% aqueous solution is higher than 100 °C. Furthermore, Triton CG-110 is viscous brown liquid, easily soluble in water and stable in highly alkaline solutions.

Propylene glycol (PPG), glycerol and polyethylene glycol 400 (PEG 400) were purchased from Sigma-Aldrich (St. Louis, MO, USA). Standards of nine main components of teat tree oil (TTO) specified in ISO 4730 and listed in Table 1 were purchased from Alfa Aesar (Heysham, Lancashire, UK) and Sigma-Aldrich (St. Louis, MO, USA). The *n*-decane used as an internal standard for GC analysis was bought from Alfa Aesar (Heysham, Lancashire, UK). For comparison, the essential oil of *M. alternifolia* commercially available was acquired from Sigma-Aldrich (St. Louis, MO, USA).

All chemicals were of reagent grade and used as received. Deionized water from a Millipore Milli-Q ultra-purification system having resistivity greater than 18.2 MΩ·cm was used in the sample preparation.

### 4.2. Extraction and Analysis of Tea Tree Oil (TTO)

The leaves and twigs of the tea tree, *Melaleuca alternifolia*, were air-dried in an air-conditioned room at 25 °C for ten days. Only leaves were used to extract TTO. Twigs are known to contain very little oil and were, thus, discarded [12]. Typically, 10 g of leaves were initially added into the round-bottom flask of the Clevenger system, in which the hydrodistillation process was proceeded over 120 min in the presence of 650 ppm Triton CG-110.

The analyses on the compositions of tea tree oil extracted by hydrodistillation were carried out either qualitatively with GC-TOF-MS or quantitatively with GC-FID. The GC-TOF-MS instrument consisted of an Agilent GC 6890N and a TOF detector (Pegasus III TOF-MS system) (Agilent Technologies, Santa Clara, CA, USA). The separation column (DB-35MS Ultra Inert, 30 mm × 25 µm × 0.25 µm) was used. The temperature profile in this GC was programmed at 100 °C for the first minute, then increased to 310 °C at the rate of 8 °C·min^−1^, and finally maintained at 310 °C for 10 min. The flow rate of helium as the carrier gas in GC-TOF-MS was kept at 1 mL·min^−1^. The operation parameters for the mass spectrometer are (1) the electron impact mode (EI) at 70 eV, and (2) the temperature of the ion source at 230 °C.

The gas chromatograph (Model GC-2014, Shimadzu Corp., Kyoto, Japan) coupled with the flame ionization detector (GC-FID) was used in quantification of nine main ingredients of tea tree oil. The HP-5 capillary column (30.0 m × 0.25 mm × 0.25 μm) was adopted as the separation column. Tea tree oil samples diluted with ethanol by 100× (*w*/*w*) was automatically injected into GC-FID with the split mode (1:100). High-purity nitrogen at a flow rate of 1 mL·min^−1^ was used as the carrier gas. The temperature program of this GC-FID was set to increase from 70 °C to 95 °C by 10 °C·min^−1^, then further to 105 °C at 1 °C·min^−1^, and finally to reach 300 °C with a heating rate of 25 °C·min^−1^. Standard solutions of nine main ingredients of tea tree oil were prepared by mixing these individual components in ethanol from their pure stocks with the purity higher than 97% in ethanol. The concentration of each component in the standard solution was in the range from 50 mg/L to 7000 mg/L. The *n*-decane with 300 mg/L was chosen as an internal standard to provide better quantitative analysis on the in-house hydrodistilled TTO.

### 4.3. Phase Diagrams

Pseudoternary phase diagrams of the mixed surfactant—tea tree oil (TTO)—water systems were constructed by both visual observation under crossed polarizers and by examination with light scattering. The formation of microemulsion was further affirmed with the measurement of aggregate size by Dynamic Light Scattering (Zetasizer Nano ZS, Malvern Panalytical Ltd., Malvern, UK).

With the phase diagrams, the appropriate concentration ranges of three components (oil, water and surfactant) resulting in the formation of microemulsion could be determined. For comparison, the TTO commercially available, in addition to the TTO in-house hydrodistilled, was used as well. Humectants, propylene glycol (PPG), glycerol and polyethylene glycol 400 (PEG 400), are commonly formulated in personal-care products and generally considered as co-surfactants. Accordingly, effect of these hygroscopic substances on the formation of microemulsion was studied as well. Notably, the commercial-grade APG surfactant always contains certain amount of water. For example, the water content in Triton CG-110 used in this work was 39.65% as measured by TGA. Hence, all compositions reported in this work were corrected duly to reflect the actual amount of each ingredients in the samples.

The samples for construction of pseudoternary phase diagrams were prepared at ambient temperature and stored in an environmental chamber at 25 °C. Specifically, the mass ratios (*S_m_*) of surfactant/co-surfactant (humectant) in mixed surfactants were selected as 0.6:1 and 1.8:1 for Trion CG-110/PPG, and 1.8:1 for both Triton CG-110/glycerol and Triton CG-110/PEG 400. In fact, the corrected Sm ratios as 0.6:1, 1.8:1 were corresponding to the ratio of 1:1 and 3:1 in preparation of sample. A ratio of 1.8:1 between Triton CG-110 and humectants was chosen due to their completely solubility.

Samples for construction of pseudoternary phase diagrams were prepared from blends with predetermined mass ratios of mixed surfactant-to-oil and, subsequently, diluted sequentially with deionized water. Typically, the samples could reach phase-equilibrium in a day. Samples showing a single phase with optical transparency, i.e., without phase-separation observed, would be subject to further instrumental analyses to determine their properties. Polarized microscopy was also applied to recognize the formation of liquid crystals in the dispersions of these pseudoternary systems.

For simplicity to locate the phase points of microemulsions, the dilution path on the phase diagram were labelled as Line x–y, in which x and y denote the relative proportion of the initial mass of mixed surfactant and the weight of oil initially present in the surfactant–oil blends, and x + y = 10. Notably, the measured mass of the mixed surfactant mentioned above was not yet corrected for water in Triton CG-110.

### 4.4. Characterization of Microemulsion

Characterization of Triton CG-110/PPG/TTO microemulsions were carried out with the measurements of electric conductivity, dynamic viscosity, and aggregate sizes.

The rheological properties of the microemulsions were assessed with a rheometer at 25 °C (Model Discovery HR-2, TA Instruments, New Castle, DE, USA). A cone-and-plate sensor (cone angle = 2° and plate diameter = 25 mm) was used. The dynamic viscosities of microemulsions with compositions on Line 3–7 were recorded with a shear rate from 1 s^−1^ to 300 s^−1^. The apparent viscosity (η) of the microemulsions with different water contents along Line 3–7 and Line 1–9 were measured in triplicate at 10 s^−1^. Besides, the viscosities of dilute microemulsion samples was also determined with the Ostwald capillary viscometers. The electrical conductivity of the microemulsions were measured at 25 ± 0.5 °C with a conductivity meter (Model CM-30G, DKK-TOA, Tokyo, Japan). Though Triton CG-110 is a nonionic surfactant, its commercial product intrinsically contained water near 40% and electrolytes as impurities, which was observed as well in other APG products [33]. Therefore, no additional electrolyte was added to the microemulsion as a conductivity marker in this work.

The average size of microemulsion aggregates as well as the polydispersity index of dispersions were analyzed by Dynamic Light Scattering (DLS) (Zetasizer Nano ZS, Malvern, UK). The He-Ne laser at 630 nm with a scattering angle of 173° was used. Measurement was performed in triplicate at 25 °C. The average droplet size was calculated from Stokes-Einstein equation using the viscosity and reflective index of microemulsion measured by Ostwald viscometers and an ABBE refractometer at 25 °C, respectively.

## Figures and Tables

**Table 1:** Compositions of the *in-house* hydrodistilled and commercial tea tree oil (TTO) by GC-FID (according to ISO 4730:2017 but with *n*-decane as an internal standard).

Component	ISO 4730 (%)	Commercial TTO (%)	As-Extracted TTO (%)
*α*-Pinene	1–4	3.0	2.5
*α*-Terpinene	6–12	9.0	8.6
p-Cymene	0.5–8	4.0	2.3
Limonene	0.5–1.5	1.7	1.3
1,8-Cineole	<10	3.0	3.6
*γ*-Terpinene	14–28	19	16.1
Terpinolene	1.5–5	3.1	2.6
Terpinen-4-ol	35–48	43.0	47.4
*α*-Terpineol	2–5	4.0	4.7
Others		10.2	10.9

## Data Availability

The data are available from the corresponding author.

## Sample Availability

Samples of the hydrodistilled tea tree oil are available from the authors.
